# RELICT-NI: Replica Detection in Synthetic Neuroimaging—A Study on Noncontrast CT and Time-of-Flight MRA

**DOI:** 10.1007/s12021-025-09745-2

**Published:** 2025-11-10

**Authors:** Orhun Utku Aydin, Alexander Koch, Adam Hilbert, Jana Rieger, Felix Lohrke, Fujimaro Ishida, Satoru Tanioka, Dietmar Frey

**Affiliations:** 1 CLAIM - Charité Lab for AI in Medicine, Charité – Universitätsmedizin Berlin, corporate member of Freie Universität Berlin and Humboldt-Universität zu Berlin, Charitéplatz 1, 101117 Berlin, Germany; 2 Department of Neurosurgery, Mie Chuo Medical Center, 2158-5 Myojin-cho, Hisai, 514- 1101 Tsu Japan; 3 Department of Neurosurgery, Mie University Graduate School of Medicine, 2-174 Edobashi, Tsu, 514-8507 Japan; 4 Department of Neurosurgery, Charité – Universitätsmedizin Berlin, Universität Berlin and Humboldt-Universität zu Berlin, Charitéplatz 1, 101117 Berlin, Germany

**Keywords:** Artificial intelligence, Deep learning, Privacy, Pattern recognition, automated, Image processing, computer-assisted

## Abstract

**Background:**

Synthetic neuroimaging data has the potential to augment and improve the generalizability of deep learning models. However, memorization in generative models can lead to unintended leakage of sensitive patient information, limiting model utility and jeopardizing patient privacy.

**Methods:**

We propose RELICT-NI (REpLIca deteCTion-NeuroImaging), a framework for detecting replicas in synthetic neuroimaging datasets. RELICT-NI evaluates image similarity using three complementary approaches: (1) image-level analysis, (2) feature-level analysis via a pretrained medical foundation model, and (3) segmentation-level analysis. RELICT-NI was validated on two clinically relevant neuroimaging use cases: non-contrast head CT with intracerebral hemorrhage (*N* = 774) and time-of-flight MR angiography of the Circle of Willis (*N* = 1,782). Expert visual scoring was used as the reference for identifying replicas. Balanced accuracy at the optimal threshold was reported to assess replica classification performance of each method.

**Results:**

The reference visual rating identified 45 of 50 and 5 of 50 generated images as replicas for the NCCT and TOF-MRA use cases, respectively. For the NCCT use case, both image-level and feature-level analyses achieved perfect replica detection (balanced accuracy = 1) at optimal thresholds. A perfect classification of replicas for the TOF-MRA case was not possible at any threshold, with the segmentation-level analysis achieving the highest balanced accuracy (0.79).

**Conclusions:**

Replica detection is a crucial but often neglected validation step in developing deep generative models in neuroimaging. The proposed RELICT-NI framework provides a standardized, easy-to-use tool for replica detection and aims to facilitate responsible and ethical synthesis of neuroimaging data.

**Relevance Statement:**

Our developed replica detection framework provides an important step towards standardized and rigorous validation practices of generative models in neuroimaging. Our method promotes the secure sharing of neuroimaging data and facilitates the development of robust deep learning models.

**Supplementary Information:**

The online version contains supplementary material available at https://doi.org/10.1007/s12021-025-09745-2.

## Introduction

Artificial intelligence in medical imaging has the potential to transform diagnostic workflows and support critical treatment decisions in fields such as radiology (Saha et al., 2024), histopathology (Dippel et al., 2024), and dermatology (Salinas et al., 2024). One of the key requirements for developing robust and generalizable deep learning models is access to large, diverse and high-quality training and validation datasets (Schwabe et al., 2024). However, the curation of such medical imaging datasets is often constrained by sensitive data sharing (Legido-Quigley et al., 2025), high acquisition costs, and limited availability of expert labeling.

To address these challenges, synthetic medical images of various modalities, anatomical regions and pathological conditions have been successfully generated using generative models like generative adversarial networks (GAN) (Ferreira et al., 2024) or diffusion-based models (DM) (Ibrahim et al., 2024; Kazerouni et al., 2023). Synthetic data can augment training sets to improve fairness, generalizability, and performance of downstream deep learning models (Frid-Adar et al., 2018; Khader et al., 2023; Khosravi et al., 2024; Ktena et al., 2024). Synthetic data should retain the predictive properties of real data and provide high quality and resolution required for many clinical applications. For optimal generative quality and utility, synthetic images are expected to be indistinguishable from real images (Park et al., 2021).

Several works used synthetic data for neuroimaging applications to increase generalizability and performance of deep learning models (Fernandez et al., 2024; Hoopes et al., 2022). A key challenge in generative modeling is the risk of reproducing real training data at varying similarity levels. While replication, i.e., synthesis of an identical copy of a training sample, is the major concern, synthetic images can also closely resemble training data without being exact copies (Akbar et al., 2023; Carlini et al., 2023). Higher levels of similarity between synthetic and real images can reduce the privacy benefits of synthetic data and decrease the added value of synthetic data augmentation by limiting diversity. This raises ethical concerns, since data that is not initially publicly shared due to data protection regulations can be unintentionally exposed by releasing a model or publishing synthetic datasets.

The use of generative models in the medical domain is a high-risk application that can jeopardize patient privacy (Giuffrè & Shung, 2023). Patient imaging data can serve as uniquely identifiable biometric information, similar to fingerprints (Packhäuser et al., 2022). This vulnerability can be exploited in adversarial attacks, such as membership inference attacks, where information about the inclusion of an individual in a training dataset can be extracted (Kuppa et al., 2021; Paul et al., 2021). Despite the existence of data protection regulations, ethical guidelines and AI research checklists (Chen et al., 2024; Tejani et al., 2024), empirical analysis of memorization in synthetic neuroimaging data generation remains largely underexplored (Ibrahim et al., 2024).

Prior works addressed content-based image retrieval (Gupta et al., 2023) and memorization in medical image generation using various image similarity measures (Dar et al., 2025). A significant gap in the field is the absence of a standardized tool and methodology for replica detection. Recent works either do not check for memorized replicas (Pan et al., 2023; Peng et al., 2023; Pinaya et al., 2022) or rely on highly task-specific, custom approaches for replica detection. For instance, Fernandez et al. calculated the overlap of real and generated labels using the Dice coefficient to find nearest neighbours (Fernandez et al., 2024), Dar et al. trained a self-supervised model to project images onto a lower dimensional embedding space and performed replica detection through correlation values (Dar et al., 2025), Packhäuser et al. train a siamese neural network to detect memorized images (Packhäuser et al., 2022, 2023), Akbar et al. used correlation of pixel intensities (Akbar et al., 2023), Aydin et al. used a predefined threshold of l2 distance ratio (Aydin et al., 2024). This inconsistency in the literature warrants a standardized, easy-to-use solution to be used as a validation step in medical image generation research.

Neuroimaging poses unique challenges for replica detection. Unlike two-dimensional scans, brain images are inherently volumetric and require specialized preprocessing—such as skull-stripping and intensity normalisation—that alters their statistical properties. Moreover, neuroimaging modalities must capture subtle, subject-specific anatomical variations and pathological changes, which may not be captured by similarity measures. Consequently, an effective replica-detection framework must be tailored and validated for three-dimensional, preprocessed volumes.

In this study, we propose a framework for identifying replicas, memorized copies of the training data, in synthetic neuroimaging datasets. Our framework evaluates image similarity using three complementary approaches: (1) image level comparison, (2) feature extraction by a medical foundation model, and (3) segmentation-level comparison. We demonstrate our framework on two clinically relevant neuroimaging use cases: generative modelling of non-contrast head CT scans with intracerebral hemorrhage and Circle of Willis arterial segments. Our standard, easy-to-use replica detection framework aims to contribute to the safe, ethical deployment of generative models in neuroimaging.

##  Methods

The data collection for this retrospective study was approved by the local Ethics Committees of following hospitals: Mie Chuo Medical Center institutional review board [permit number: MCERB-202321], Matsusaka Chuo General Hospital institutional review board [permit number: 325], Suzuka Kaisei Hospital institutional review board [permit number: 2020-05], and Mie University Hospital institutional review board [permit number: T2023-7]. Written informed consent was waived due to the retrospective nature of the analysis.

### Use Cases

We first describe the use cases that generated the synthetic neuroimaging data used in this study. Each use case includes: a real training dataset, the generative model and the corresponding segmentation model that segments relevant structures or pathologies depending on the use case.

#### Use case 1: 3D NCCT with Intracerebral hemorrhage

In use case 1, the image generation task was to synthesise 3D non-contrast computed tomography (NCCT) data with intracerebral hemorrhage (ICH) as the leading pathology. The training dataset included 387 patients with baseline and follow up NCCT imaging within 24 h (in total 774 images) with the primary diagnosis of ICH from 4 hospitals in Japan: Mie Chuo Medical Center, Matsusaka Chuo General Hospital, Suzuka Kaisei Hospital and Mie University Hospital. The NCCT volumes were registered to the MNI space, with a resolution of 182× 218 × 182 voxels and voxel spacing of 1 × 1 × 1 millimeters. Detailed patient characteristics and dataset information have been previously reported (Tanioka et al., 2024).

A latent diffusion model architecture was used for generative modelling. Latent embeddings were produced using a vector-quantized autoencoder with residual-vector quantization, resulting in 8x downsampling of the original data dimensions. The elucidated diffusion training method was used for training and synthetic images were generated using a DPM-Solver + + for 100 sampling steps (Karras et al., 2022).

An nnUnet segmentation model was trained on the training set of 774 scans with manually segmented binary ICH labels (Isensee et al., 2021). The model was trained for 100 epochs using the default nnUnet hyperparameters, with CT normalization and 5 fold cross validation.

The open source implementation of the diffusion model is available at:

https://github.com/claim-berlin/relict.

#### Use case 2: 3D TOF-MRA

In use case 2, the image generation task was to synthesise healthy 3D time-of-flight magnetic resonance angiography (TOF-MRA) data. A 3D adaptation of the StyleGANv2 architecture was used for generative modelling of the Circle of Willis (Karras et al., 2020). The training data was open source and consisted of 1782 3D TOF MRA volumes from 7 different datasets as detailed in a prior work (Aydin et al., 2024). Preprocessing included registration to a TOF-MRA template and cropping to 128 × 128 × 32 voxels with 0.62 × 0.62 × 0.62 millimeters voxel spacing, centered around the Circle of Willis.

An nnUnet segmentation model was trained on 50 patients from the TopCoW (Topology-Aware Anatomical Segmentation of the.

Circle of Willis for CTA and MRA) dataset to segment Circle of Willis artery segments in a multiclass setting (Isensee et al., 2021; Yang et al., 2024). Paired artery segment labels were merged to create a single class, resulting in following artery segments: Internal carotid artery (ICA), basilar artery (BA), posterior communicating artery (Pcom), anterior communicating artery (Acom), the posterior cerebral artery (PCA), anterior cerebral artery (ACA), and the first segment of the middle cerebral artery (M1). The model was trained for 1,000 epochs using the default nnUnet hyperparameters, with MR normalization and 5 fold cross validation.

Details on the generative model architecture, hyperparameters, and open-source implementation can be found at:

https://github.com/claim-berlin/3D_StyleGAN_Circle_of_Willis.

### Replica Detection

#### Definition

The definition of replica in scientific literature is ambiguous, and task-dependent (Dar et al., 2025; Fernandez et al., 2023). In this work, we define replicas as synthetic images that are identical to real images in the training set with respect to anatomical or pathological image features.

#### Assessing Image Similarity

The similarity between a synthetic and real neuroimaging volume can be analyzed from different perspectives. First, volumes can be compared directly, i.e., voxel by voxel. Second, a more abstract comparison can be made by compressing images or extracting image features. Third, only relevant portions of the images can be compared by utilizing segmentation models trained to identify the regions of interests. To robustly identify potential replicas in medical imaging, our framework uses these three complementary perspectives. First, the image-level analysis directly compares voxel intensities using the following measures: mean absolute error (MAE), root mean square error (RMSE), and structural similarity index measure (SSIM) (Wang et al., 2004). Second, the feature-level analysis uses a pretrained medical foundation model to extract feature representations from the images (Chen et al., 2019). These feature embeddings are compared using RMSE and cosine similarity to capture medically relevant differences that may not be evident from raw pixel values. Finally, the segmentation-level analysis focuses on clinically significant regions of interest by comparing segmentation masks, such as those delineating hemorrhage lesions in NCCT images, using the Dice coefficient (Dice, 1945) and average surface distance (ASD) (*GitHub - Google-Deepmind/Surface-Distance: Library to Compute Surface Distance Based Performance Metrics for Segmentation Tasks.*, n.d.). Detailed descriptions of the individual analyses levels and used measures are provided in Appendix 1-A.

#### Overview of Study Design

Our analysis using the proposed replica detection framework consisted of the following steps: (1) a generative model is trained on 3D neuroimaging data, (2) the trained generative model is used to generate a synthetic dataset, (3) for each synthetic image, training images are ranked based on similarity to identify the closest training image, (4) an expert visual scoring is performed to score similarity of each synthetic image and closest training image leading to a binary replica decision, (5) the various measures are tested at their optimal thresholds for their replica detection performance.

**Fig. 1:**
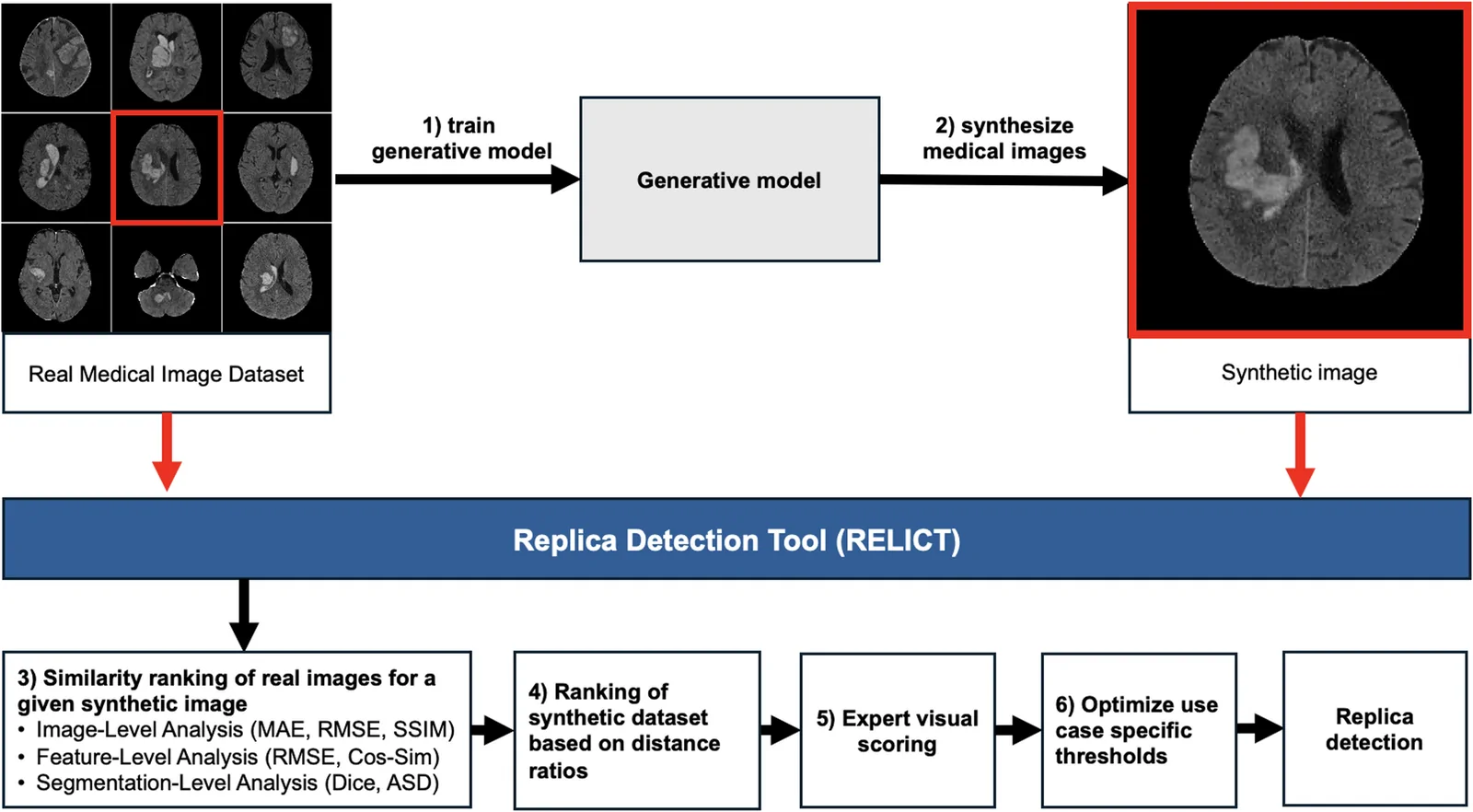
Overview of the replica detection framework

For subsequent automatization of replica detection in future neuroimaging studies, we propose following steps: steps 1–3 remain the same, 4) ranking of synthetic images within the synthetic dataset by their distance ratios for each measure, 5) visual scoring of a small subset of highest likely replica images with lowest distance ratios 6) defining use case specific thresholds for automated replica detection using visual scoring results. The methodological overview of the RELICT-NI framework is shown in Fig. 1.

#### Distance Ratio and Replica Decision

In our replica detection framework, we use the methodology introduced by Carlini et al. and Yoon et al. (Carlini et al., 2023; Yoon et al., 2023). This approach first computes a measure, such as RMSE, between the generated image under evaluation and all images in the training set. Second, the training images are sorted from most similar to least similar and the closest training image is identified. Third, we use following equation to compute the distance ratio by comparing a synthetic image to real images:

$$M(\widehat{x},x;S_{\widehat{x}})=\frac{M(\widehat{x},x)}{E_{y\in S_{\widehat{x}}}[M(\widehat{x},y)]}$$

for a given distance measure M, where $\:\widehat{x}$ is the synthetic image under evaluation for replica detection, x is the closest image in the training set, S$\:\widehat{x}$ is the subset of n closest images in the training set to $\:\widehat{x}$. This equation computes the measure value for the closest training image x and divides it by the mean value of the n closest training images. This equation is modified from the work by Carlini et al. All experiments were performed with *n* = 50 closest training images for S$\:\widehat{x}$.

The distance ratio provides information about how “abnormally close” the synthetic image is to the closest training image (Carlini et al., 2023). A binary decision whether the synthetic image is a replica can be made by thresholding the distance ratios. Here, the threshold T might depend on image properties (e.g. intensity range, image variation within training set) and should ultimately reflect the user’s tolerance for resemblance:

$$Replica\;Decision=\begin{cases} Replica,\,if\,M\,(\widehat{x},x;S_{\widehat{x}}) < T, \\Not\;a\;Replica,\,if\,M\,(\widehat{x},x;S_{\widehat{x}}) \geq T.\end{cases}$$

Equation 1 based on the distance ratio was used for the image-level and feature-level analysis. To ensure consistency when applying threshold comparisons, similarity measures were converted into distance-based measures by first normalizing the values between 0 and 1 and subtracting their values from 1 (e.g., a Dice coefficient of 0.7 was transformed into a distance of 0.3).

For the segmentation-level analysis only the absolute value of the segmentation evaluation result was used for replica detection instead of the ratio in Eq. 1. This decision was based on the consideration that the segmentation step already isolates the foreground region of interest and disregards background information successfully.

### Visual Scoring of Replicas

For each use case, 50 synthetic images were generated. Since visually comparing all synthetic images to each training image was infeasible, each synthetic image was paired with its most similar training image, calculated by RMSE. This preliminary step allowed a more detailed and reliable image comparison within feasible efforts of clinical raters.

**Fig. 2:**
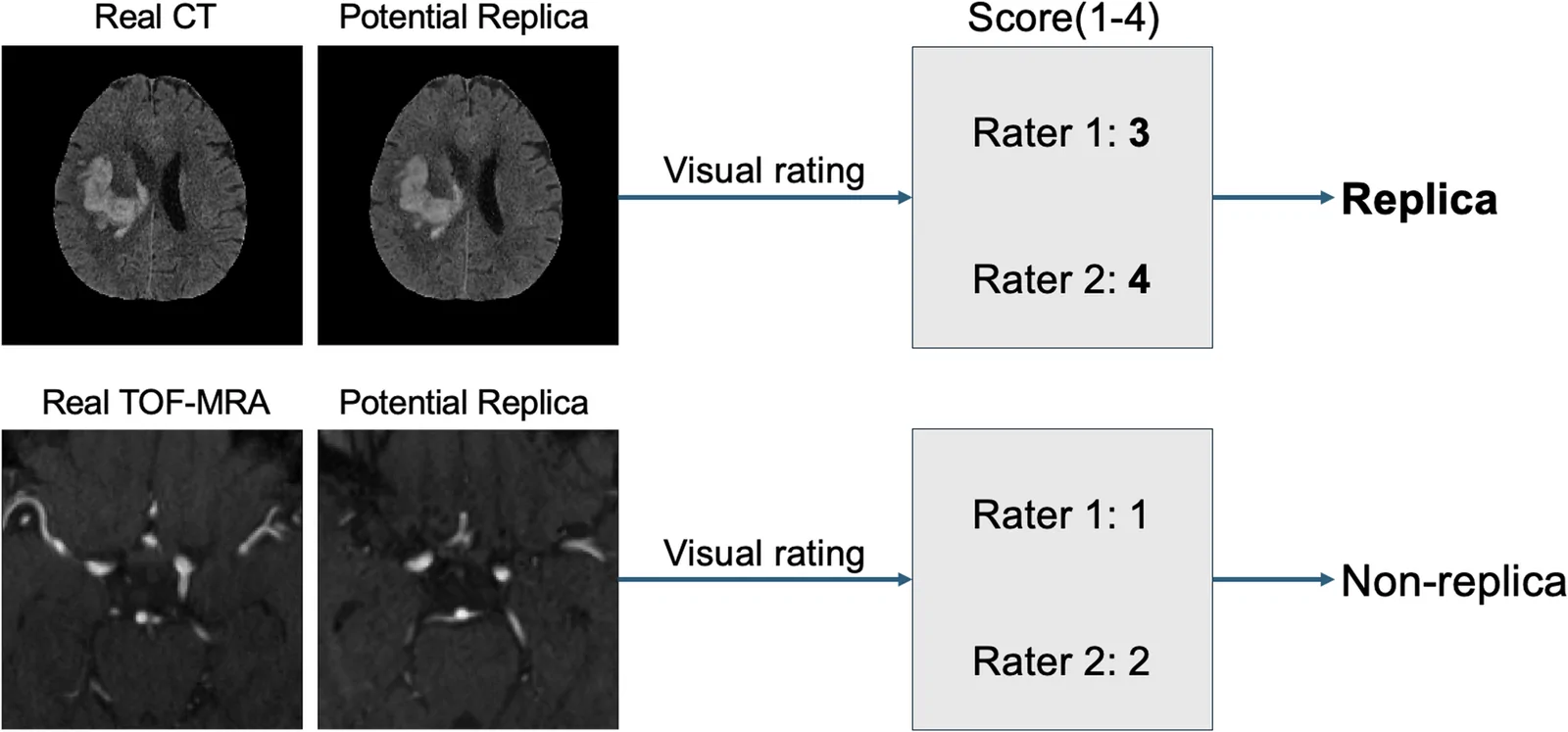
Subjective visual scoring examples for two pairs of real and generated images

Two senior raters independently inspected each synthetic-real image pair using the predefined subjective visual scoring criteria (Table 1). The visual scoring was performed using ITK-SNAP by inspecting the pair of side by side synthetic and real image side by side (Yushkevich et al., 2006). A 4 point Likert-type scale (Likert, 1932) was chosen to avoid neutral decisions. Visual scores of 3 or 4 led to the classification of a synthetic image as a replica. In cases where the raters disagreed on the replica classification decision, the images were re-evaluated by each rater individually. The visual scoring is illustrated with examples in Fig. 2.

**Table 1:** Subjective visual scoring for replica detection ground truth creation. The scoring is performed using a Likert-type scale where increasing scores reflect the rater’s subjective confidence that an image is a replica

Scale	1	2	3	4
**Point**	**Certainly not a Replica**	**Probably not a replica**	**Probably a Replica**	**Certainly Replica**
**Description**	Two different images, no resemblances	Images are different, with considerable differences in anatomy, pathology or background	Images are very similar, with some minor differences in anatomy, pathology or background	Images are mostly identical

### Software

The framework is written in the Python programming language (Version: 3.10) and implements the image comparison measures from open-source libraries. The authors will make efforts to assess ongoing research in the field and aim to keep the repository updated with successful replica detection methods as they are developed. Additionally, we welcome contributions from the open-source community to expand the framework.

Computation was performed on the HPC for research cluster of the Berlin Institute of Health using 50 CPU cores and a single V100 GPU. The replica detection code is available open-source in the following GitHub repository:

https://github.com/claim-berlin/relict.

### Evaluation

We showcase the proposed application of RELICT-NI in the two presented use cases. First, for each analysis-level, replica detection thresholds were analyzed by using the visual scoring ground truth and systematically evaluating performance across 0.01 increments of the thresholds relative to the value of the measure. The replica detection performance was reported using balanced accuracy to equally consider sensitivity and specificity. Next, we surveyed the results of each metric and determined if an adequate separation of replicas was achieved by any threshold value.

Synthetic datasets have the highest potential when generated at large sample sizes, therefore automation of replica detection is desired. To this end, we further evaluate RELICT-NI for automated replica detection on an independent, larger sample. First, we determined the threshold maximizing separation of replicas based on the visual scoring.

Specifically, the threshold was set to the mean value of the replica with the highest and the non-replica with the lowest distance ratio. Second, we generated an additional 1000 synthetic samples and test generalization of this threshold. Lastly, we plotted a histogram of distance ratios and visually inspected synthetic images from both sides of the selected threshold.

The runtime was reported in minutes for each analysis method, using a typical workstation computer.

## Results

### Visual Rating Results

**Fig. 3:**
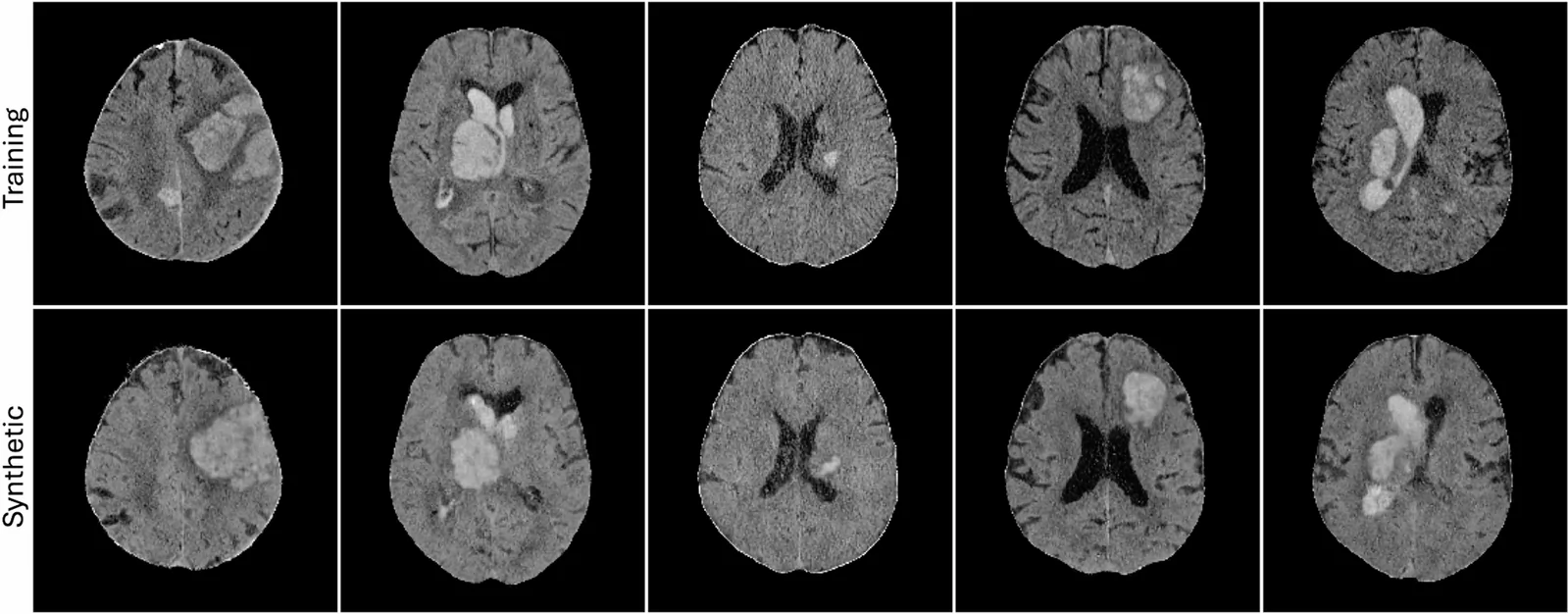
NCCT images classified as non-replicas based on visual rating

**Fig. 4:**
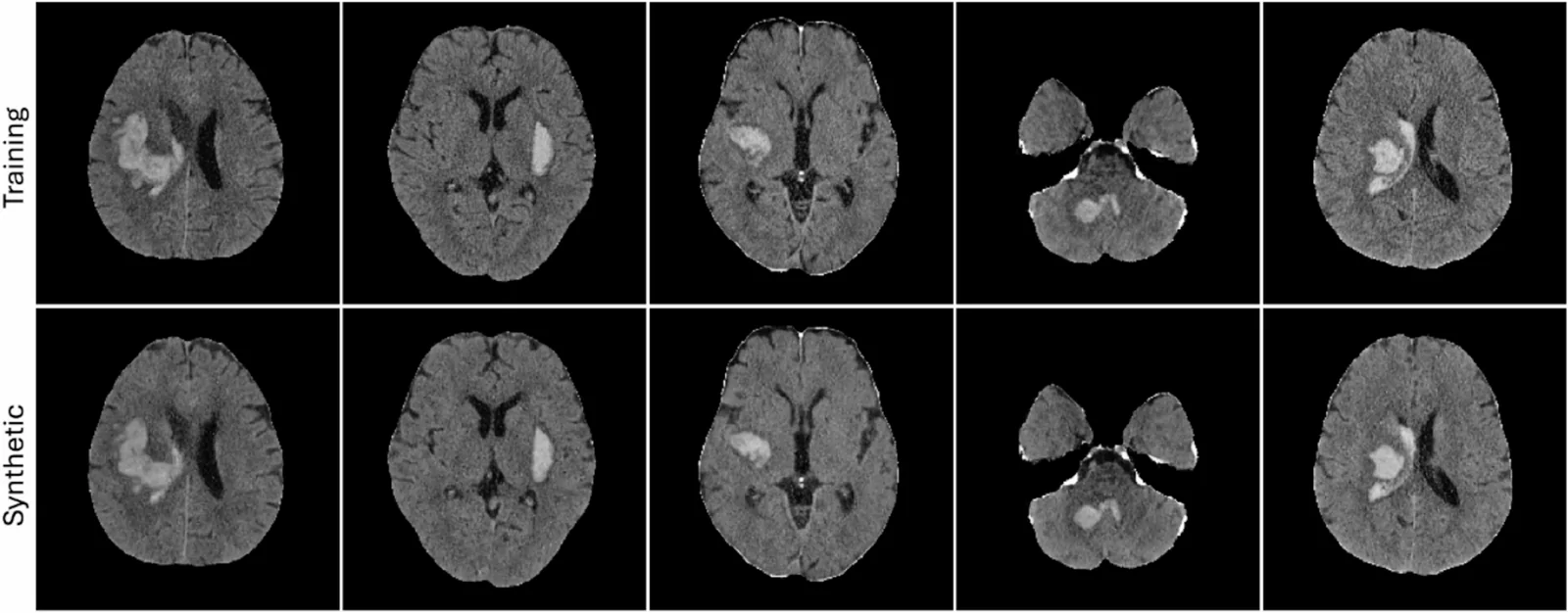
NCCT images classified as replicas based on visual rating

**Fig. 5:**
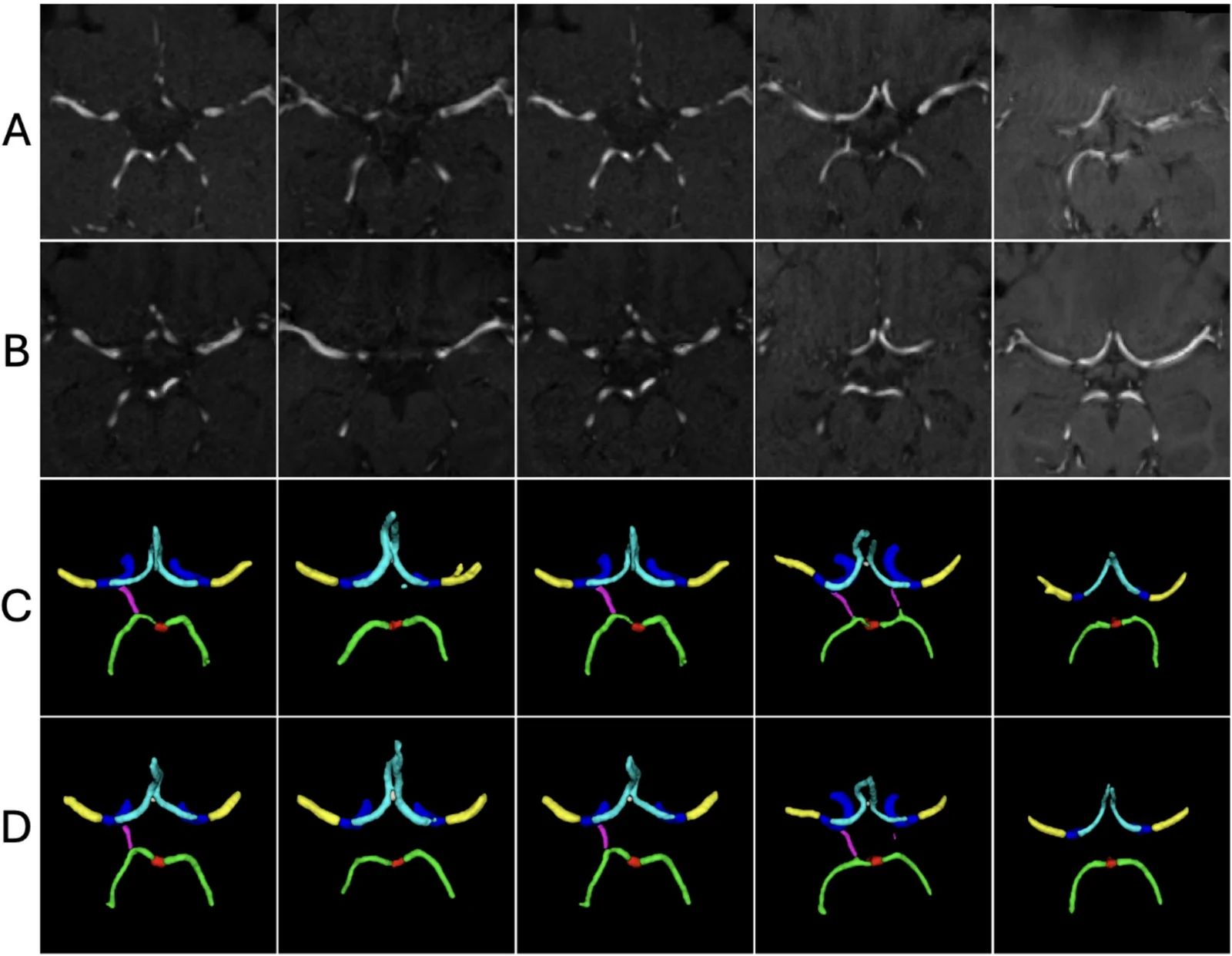
TOF-MRA images classified as replicas based on visual rating. (**A**) Real TOF-MRA data, (**B**) synthetic TOF-MRA data, (**C**) segmentations of real volumes from row A, (**D**) segmentations of synthetic volumes from row B

In the NCCT use case, raters identified 45 out of 50 synthetic images as replicas, with the remaining 5 classified as non-replicas. In the TOF-MRA use-case, 5 images were identified as replicas and 45 as non-replicas. The raters agreed in the replica detection decision in 46 out of 50 cases (92%) for the NCCT use case and 41 out of 50 cases (82%) for the TOF-MRA use case. The median visual score was 3 and 4 for the NCCT images and 1 and 2 for the TOF-MRA images for the two raters respectively. Figs 2, 3, 4 , present example pairs of real and synthetic images assessed by the raters Fig. 5.

### Replica Detection

**Fig. 6:**
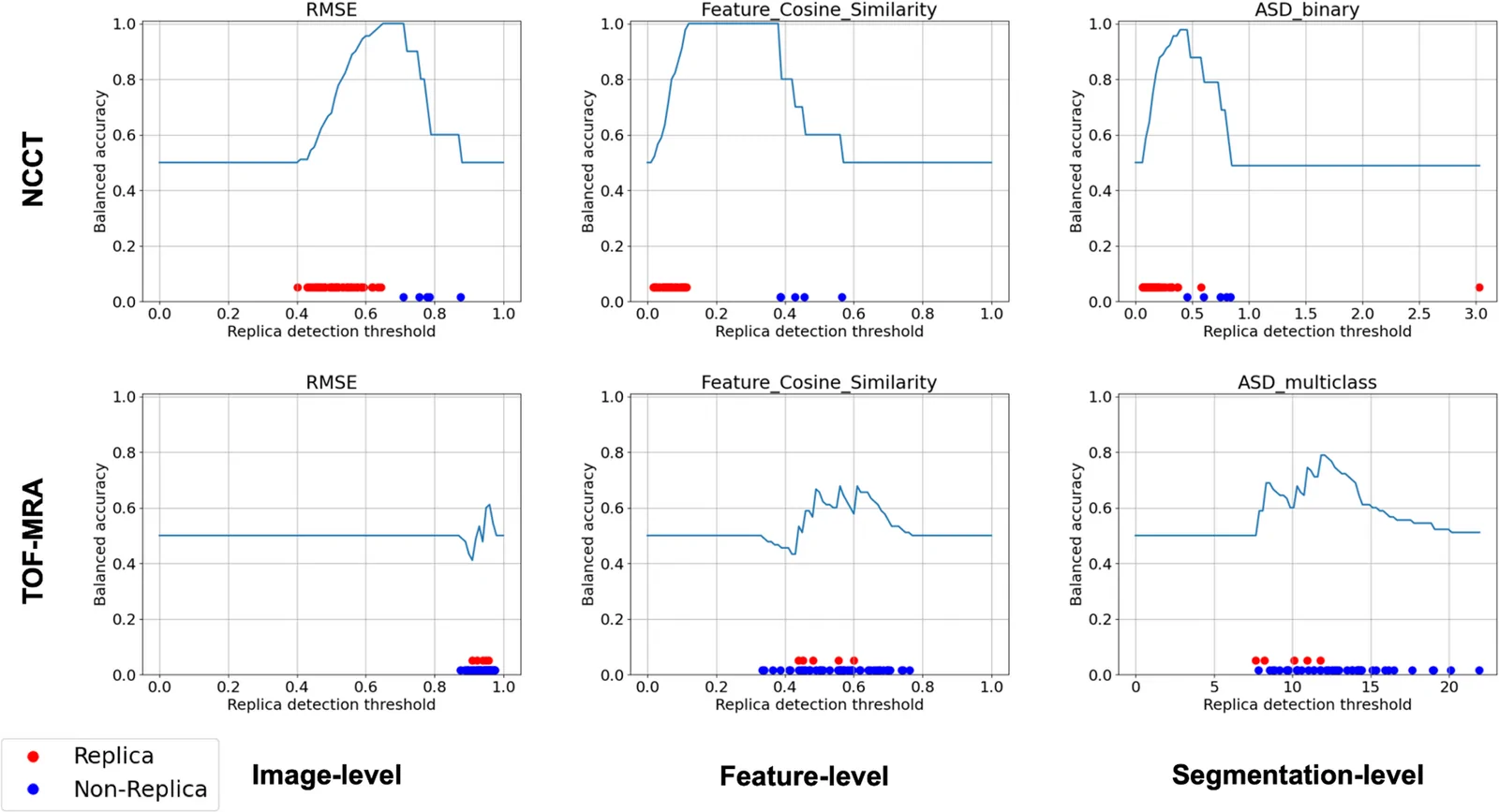
Quantitative evaluation of replica detection performance for NCCT and TOF-MRA use cases

The optimal threshold for replica detection varied based on the analysis-level and measure used (Fig. 6, Appendix 1B). For the NCCT use case, all measures tested on the image-level and feature-level analysis allowed for finding an optimal threshold to identify replicas. This indicated a perfect alignment with the visual rating (balanced accuracy of 1). The segmentation-level measures Dice and ASD had lower balanced accuracies of 0.96 and 0.98 respectively. The closest images identified by RMSE based preselection were also identified by all other measures as the closest image in 47 out of 50 images.

**Fig. 7:**
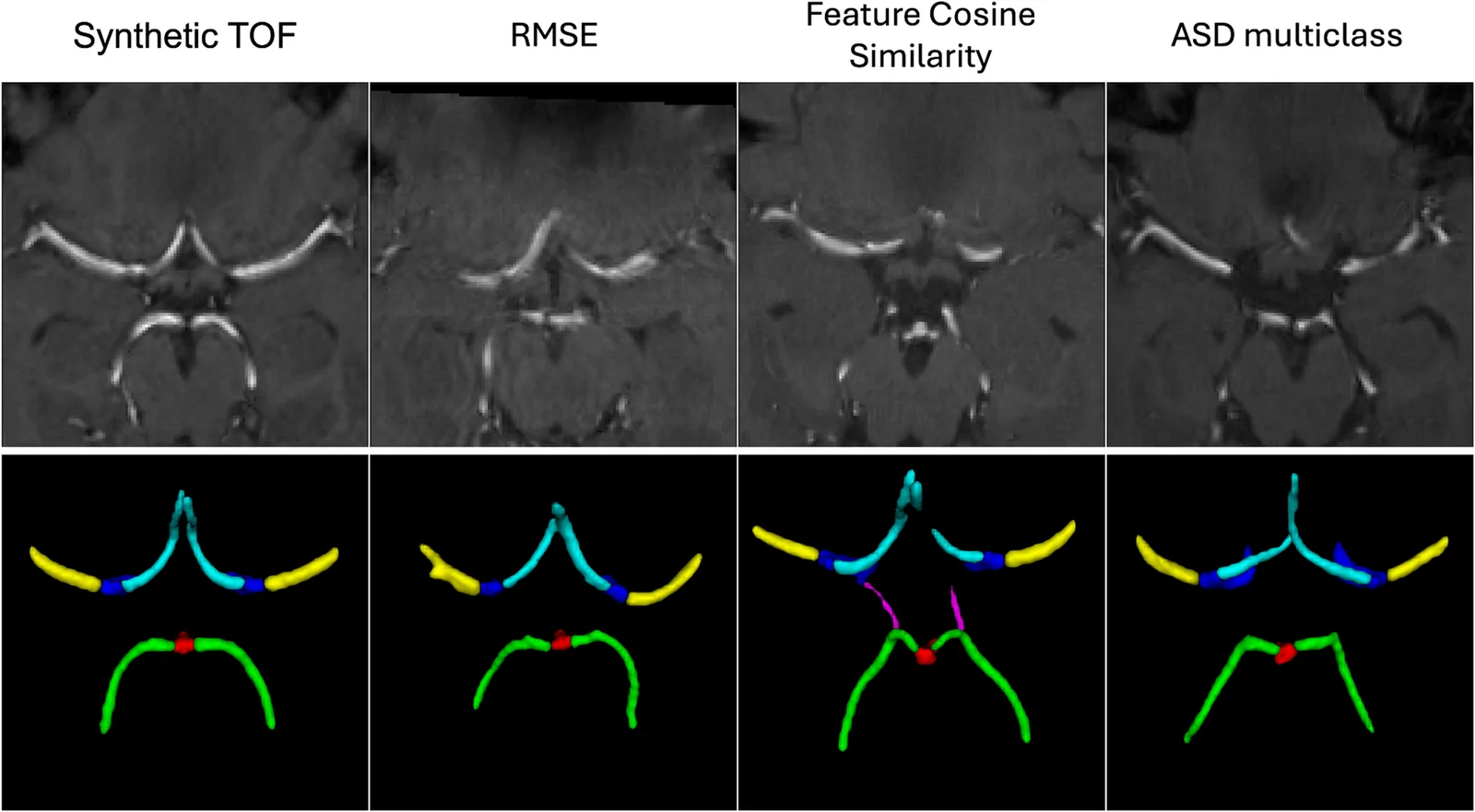
Closest training images of generated TOF images from the training dataset selected by various measures

In the TOF-MRA use case, the analysed measures ranked training images differently for all 50 synthetic images and thus identified different images as closest, compared to the preselection method (Fig. 7). An example synthetic image with different closest training images identified by RMSE, feature cosine similarity and multiclass ASD are shown in Fig. 7. The segmentation-level ASD measure achieved the highest replica detection accuracy, with a balanced accuracy of 0.8 in binary and 0.79 in multiclass settings. All analysed image-level and feature-level measures resulted in a balanced accuracy lower than 0.72.

An overview of all used measures with their respective analysis levels and runtimes can be found in Appendix 1-A, Table 2.

**Fig. 8:**
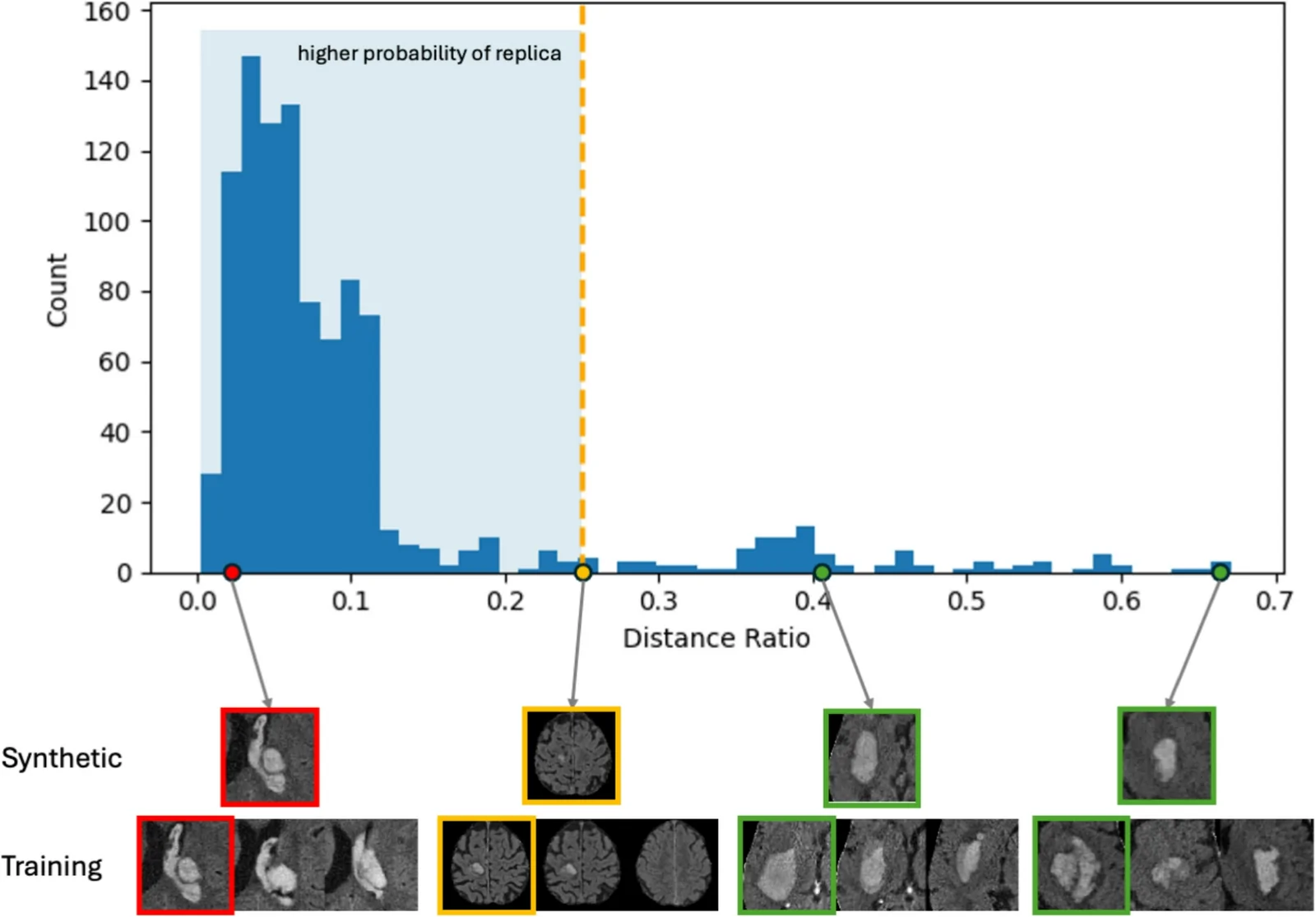
Histogram of distance ratios based on feature-level analysis. 1000 synthetic NCCTs were analyzed. Feature vectors extracted using MedicalNet were compared with cosine similarity. The threshold at 0.25 is shown with yellow dashed lines. Each synthetic image (Synthetic) is shown with the closest 3 images from the training set (Training). The closest training image has a coloured frame. The selected threshold flags 905 out of 1000 synthetic images as replicas. The red frame indicates replicas, the yellow frame indicates examples at the replication detection threshold, and the green frame indicates non-replicas

Overall, the visual rating allowed us to identify a replica detection threshold that separates replicas from non-replicas in the NCCT use case, whereas there was no perfect threshold to separate the replicas in the TOF MRA use case. Based on the visual scoring, feature cosine similarity presented the most robust separation and allowed for selection of a threshold for automation. A threshold value of 0.25, maximizing the margin between the synthetic replica with the highest distance ratio (0.11) and the non-replica with the lowest distance ratio (0.39) was selected (Fig. 6, NCCT, feature-level, cosine similarity). This threshold showed robust generalization on further 1000 synthetic cases and flagged 905 out of 1000 cases as replicas, similarly to the visually scored subset where 10% were identified as replicas. As shown on four examples in Fig. 8, the selected threshold generalized well to the larger sample.

## Discussion

We propose a replica detection framework as a standard validation step in neuroimaging generation research. Memorization in generative models and resulting replicated images can be detected using image-level, feature-level or segmentation-level comparisons. Our research confirms previous reports of memorization from the medical and natural image generation studies also for neuroimaging generation use cases, and aims to raise awareness about the significant risks posed by this underexplored issue.

Beyond confirming the threat of image replication, our analysis provided additional insights. Visual rating revealed a clear discrepancy in replica percentages between the two use cases: 90% of generated NCCTs were classified as replicas, compared to only 10% in TOF-MRAs. The image-level and feature-level analysis agreed best with the visual scoring in the NCCT use case, reflecting the near-identical appearance of the volume pairs. In contrast, for the TOF-MRAs, the segmentation-level analysis outperformed the image- and feature-level methods. This is likely due to images showing more abstract similarities, i.e. in vessel anatomy, bifurcations, that are not fully captured by image or feature level comparisons. Furthermore, we demonstrated the ranking of synthetic images based on their distance ratios, enabling the identification of replica candidates automatically. Decision thresholds can be set and adjusted via visual assessment and similarity criteria to enable automated detection in future synthetic datasets.

Memorization is a major concern and has implications for model development, downstream synthetic data usage, and sharing. It is increasingly considered during generative model development, and factors contributing to memorization are being explored to develop mitigation strategies (Dombrowski et al., 2024; Dutt et al., 2024; Somepalli et al., 2023). Research in natural image generation shows that models trained on smaller datasets are more prone to memorization. Additionally, it has been reported that larger, more complex models memorize faster (Tirumala et al., 2022). In the NCCT use case, we trained a large diffusion-based model on a small dataset of 774 high resolution 3D images. The dataset also contained baseline and follow-up images of the same patients, which may have increased memorization risk, as data duplication has also been reported to increase memorization (Carlini et al., 2023).

Recently, differential privacy (DP) has been proposed for generative image modelling to share sensitive patient data with privacy guarantees. DP is an active area of research and allows a tradeoff between data utility and privacy preservation. By decreasing the parameters (epsilon, delta), it is possible to obtain a stronger privacy guarantee, at the cost of reducing the utility of the data. Thus, practitioners must balance the risks of leaking sensitive data with high utility. Especially for diffusion based models the implementation of DP is practically challenging (Dockhorn et al., 2023). We see this as an important example application of our replication detection pipeline as a filter to additionally safeguard sharing data with potential replication of patient information. Since DP can not guarantee that no sensitive data is leaked, it is also not a panacea to avoid legal constraints in data sharing.

Generated data is increasingly used for medical data augmentation (Ktena et al., 2024). Memorized images are unlikely to benefit downstream tasks since they do not provide additional data diversity. We hypothesize that our framework can filter valuable, unique images from generated datasets. Data augmentation using unique images can help address the data diversity problem in medical deep learning models (Hofmanninger et al., 2020). With this replica filter, even predominantly memorizing generative models, as in the NCCT use case, can be evaluated for downstream use.

Based on our findings we have several recommendations for using RELICT-NI for replica detection. First, the optimal replica detection threshold varies by dataset and comparison measure. Rather than relying on a single threshold, we recommend using the proposed framework to identify pairs of synthetic and real data that are most likely to be replicas. This information is an output of the framework where pairs are ranked based on either their distance ratios or segmentation measure values. In a separate step, the ranking of likely memorized images should be manually reviewed to fine-tune replica detection thresholds if automation is desired. We show how selecting a threshold based on visual scoring on a small subset of synthetic samples and applying it on a larger independent set can enable automation. Second, the training setup of the generative model should be considered during replica detection. Especially if the generative model was trained using data augmentations, RELICT-NI cannot guarantee to find the closest real image, since augmentations are not considered explicitly. In this case, feature-level analyses can be explored such as training of self-supervised models using contrastive learning for replica detection for individual datasets (Dar et al., 2025), although these approaches require training of a dataset specific model. Alternatively, replica detection methods that increase robustness towards variations caused by data augmentation could be explored for neuroimaging studies (Somepalli et al., 2023). Third, image-level analysis can be computationally expensive for large training datasets. In such cases, cosine similarity of embeddings using established medical foundation models, such as MedicalNet, should be preferred. Future framework improvements could incorporate more recent foundation models like BiomedParse (Zhao et al., 2025) or MedSAM (Ma et al., 2024).

Several checklists and reporting guidelines aim to ensure reproducibility and transparency of medical AI research and to ensure reliability of published scientific evidence (Lekadir et al., 2025; Tejani et al., 2024). However, current guidelines primarily focus on predictive AI performance and reporting, with limited adaptation for potential challenges of generative models. Our results suggest that a standardised replica detection framework can identify replicas in synthetic datasets offering insights into dataset reliability and quality. We advocate for increased awareness of synthetic data sharing risks, and the inclusion of replica detection requirements in generative AI reporting guidelines.

Our study has several limitations. First, the proposed replica detection framework was tested in a limited number of neuroimaging use cases, each using a single generative modelling approach, preventing direct comparisons of memorization across model architectures. This was due to the significant hyperparameter tuning and computational resources required for successful generative model training. Generalization of the proposed framework to other neuroimaging modalities, such as functional MRI or PET-CT should be explored in future studies. Second, only a limited number of image comparison measures were used and a single medical foundational model was tested due to the exploratory nature of our replica detection tool. Third, in the subjective visual rating, the two senior raters assessed only the closest image suggested by a single measure, RMSE, because pairwise comparison of all images in the training set was not feasible due to time constraints. Fourth, the reported maximum replica detection accuracy is based on visual expert scoring as ground truth which allowed us to find optimal thresholds. Generalization of these thresholds across diverse datasets needs to be assessed in further studies however this step requires significant efforts with respect to generative modelling and visual assessment of synthetic images.

## Conclusion

Replica detection is an important, but often neglected quality assurance step for validation of generative models in medical imaging. Standardized replica detection methods need to be developed and included in checklists for AI-based studies in neurology, radiology and medicine in general to ensure robustness for medical application of generative AI. Identifying memorization and preventing duplication of training data are crucial for preventing patient privacy violations of generative models. Our developed framework provides an important step towards standardized and rigorous validation practices of generative models with potential for safer sharing of synthetic neuroimaging data.

## Supplementary Information

Below is the link to the electronic supplementary material.

Supplementary Material 1

## Data Availability

The proposed framework is available open-source in the following github repository: https://github.com/claim-berlin/relict The generative models and training code from the two use cases are available open source: Use case 1: https://github.com/claim-berlin/relict/tree/main/brain-ae Use case 2: https://github.com/claim-berlin/3D_StyleGAN_Circle_of_Willis Imaging data are available through the corresponding author upon reasonable request for use case 1 and gathered from open-source datasets as detailed in the referenced article Aydin et al 2024 for use case 2.

## Abbreviations

3D: Three-Dimensional
ACA: Anterior Cerebral Artery
Acom: Anterior Communicating Artery
AI: Artificial Intelligence
ASD: Average Surface Distance
BA: Basilar Artery
CNN: Convolutional Neural Network
CT: Computed Tomography
DP: Differential Privacy
DM: Diffusion-based Models
GAN: Generative Adversarial Network
GPU: Graphics Processing Unit
HPC: High-Performance Computing
ICH: Intracerebral Hemorrhage
ICA: Internal Carotid Artery
MAE: Mean Absolute Error
MNI: Montreal Neurological Institute
MR: Magnetic Resonance
MRA: Magnetic Resonance Angiography
M1: First Segment of the Middle Cerebral Artery
PCA: Posterior Cerebral Artery
Pcom: Posterior Communicating Artery
RMSE: Root Mean Square Error
ROI: Region of Interest
SSIM: Structural Similarity Index Measure
TOF-MRA: Time-of-Flight Magnetic Resonance Angiography
TopCoW: Topology-Aware Anatomical Segmentation of the
