# Supplementary material for: RELICT-NI: Replica Detection in Synthetic Neuroimaging—A Study on Noncontrast CT and Time-of-Flight MRA
Source: Neuroinformatics. 2025 Nov 10;23(4):54. doi: 10.1007/s12021-025-09745-2 (PMC12602640; doi:10.1007/s12021-025-09745-2)
Supplement: Supplementary file 1 — Supplementary Material 1 [file 12021_2025_9745_MOESM1_ESM.docx]

# Supplementary Materials

**Appendix 1.**

**A. Measures for image comparison**

**Image-level Analysis**

In the image-level analysis voxels constituting the synthetic and real image are directly compared based on various measures to compute a distance or similarity score. Image-level analysis constitutes a simple and explainable method to compare images and is adopted in previous works for replica detection tasks (Carlini et al., 2023; Yoon et al., 2023). In our work, we use the following measures for image-level analysis: mean absolute error (MAE), root mean square error (RMSE) and structural similarity index measure (SSIM) (Wang et al., 2004).

**Mean Absolute Error**The Mean Absolute Error (MAE) is calculated as the average of the absolute differences between corresponding voxels in the generated and real volumes:

[
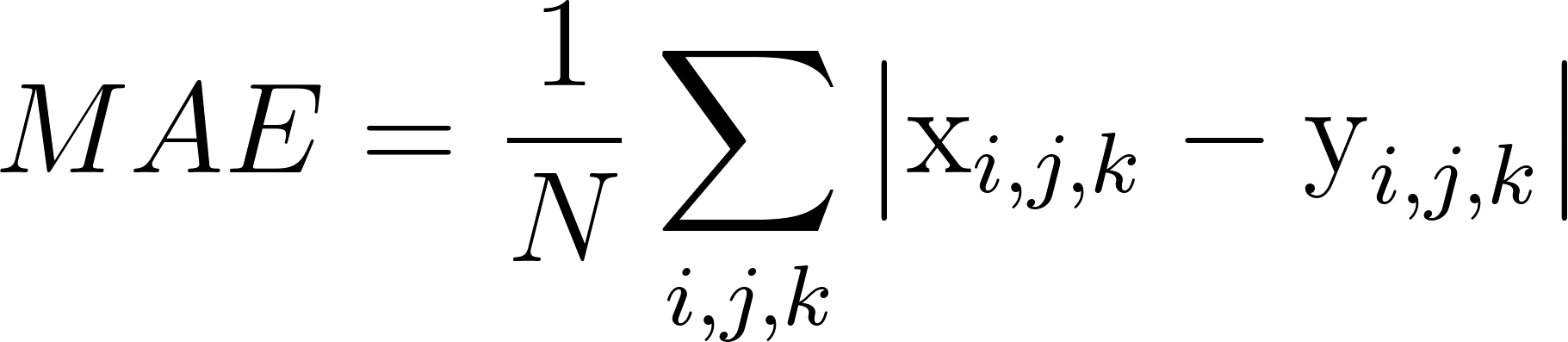
](https://www.codecogs.com/eqnedit.php?latex=MAE%20%3D%20%5Cfrac%7B1%7D%7BN%7D%20%5Csum_%7Bi%2Cj%2Ck%7D%20%5Cleft%7C%20%5Ctext%7Bx%7D_%7Bi%2Cj%2Ck%7D%20-%20%5Ctext%7By%7D_%7Bi%2Cj%2Ck%7D%20%5Cright%7C#0)

where N is the total number of voxels.

**Root Mean Squared Error**The Root Mean Squared Error (RMSE) is calculated as the square root of the average of the squared differences between corresponding voxels in the generated and real volumes:

[
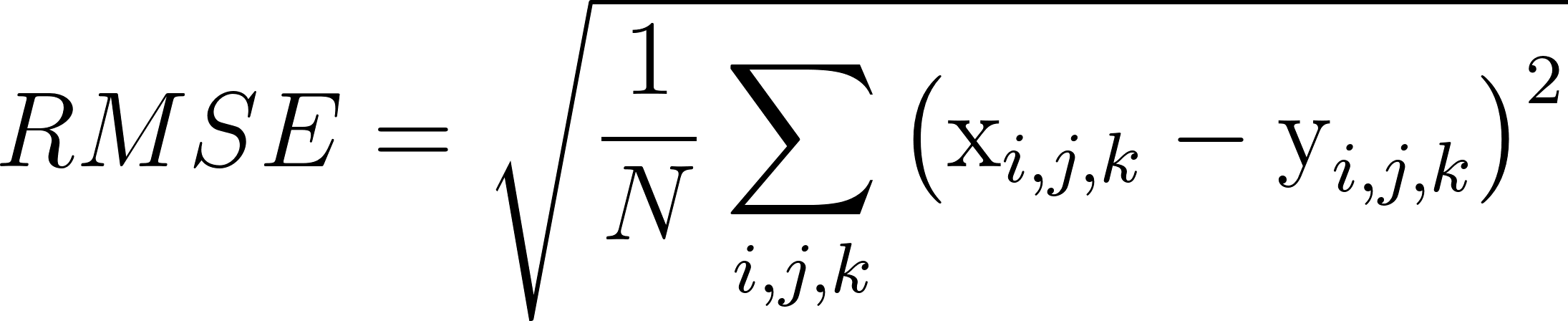
](https://www.codecogs.com/eqnedit.php?latex=RMSE%20%3D%20%5Csqrt%7B%5Cfrac%7B1%7D%7BN%7D%20%5Csum_%7Bi%2Cj%2Ck%7D%20%5Cleft(%20%5Ctext%7Bx%7D_%7Bi%2Cj%2Ck%7D%20-%20%5Ctext%7By%7D_%7Bi%2Cj%2Ck%7D%20%5Cright)%5E2%20%7D#0)

**Mean Structural Similarity Index Measure**

The SSIM was calculated using the structural_similarity function of the skimage.metrics library (version=XXX) based on the implementation by Wang et al using a gaussian weighting function with a standard deviation of 1.5 in the calculation of SSIM (Wang et al., 2004). The SSIM considers the luminance, contrast and structure of the images. To provide a single measure for comparison between a pair of real and generated images the mean SSIM was used for replica detection (Zhou Wang & Bovik, 2009).

[**
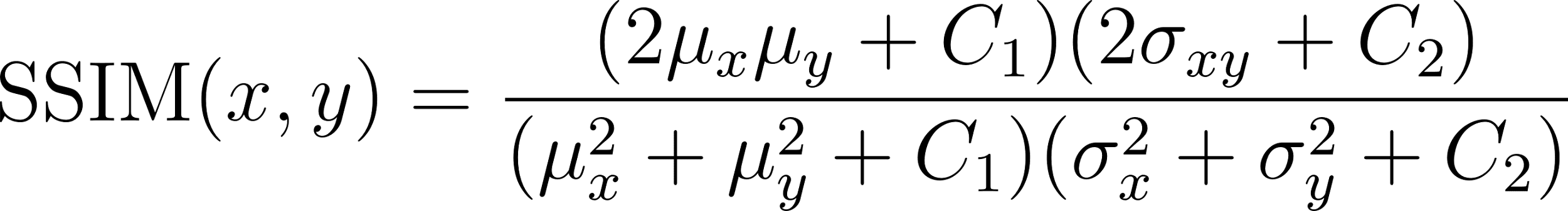
**](https://www.codecogs.com/eqnedit.php?latex=%20%5Ctext%7BSSIM%7D(x%2Cy)%20%3D%20%5Cfrac%7B(2%5Cmu_x%20%5Cmu_y%20%2B%20C_1)(2%5Csigma_%7Bxy%7D%20%2B%20C_2)%7D%7B(%5Cmu_x%5E2%20%2B%20%5Cmu_y%5E2%20%2B%20C_1)(%5Csigma_x%5E2%20%2B%20%5Csigma_y%5E2%20%2B%20C_2)%7D%20#0)

Where μx, μy are the pixel mean values, σx, σy are the standard deviations, σxy the covariance of x and y, and C1, C2 are stabilisers.

**Feature-level Analysis**

Feature-level analysis aims to reduce the dimensions of the original images by using a pretrained encoder, and subsequently comparing feature representations instead of the original voxel or pixel values. The feature-level comparison has been shown to allow a more medically relevant evaluation of image similarity (Dar et al., 2025; Gupta et al., 2023; Jush et al., 2024). In our work we use the pretrained Resnet-50 MedicalNet as a medical foundation model for feature extraction (S. Chen et al., 2019). The network has been trained on 23 medical image segmentation datasets and has been used in other works to extract feature representations from medical images (S. Chen et al., 2019; Tak et al., 2024). Feature-level analysis can be computationally faster especially if GPU resources are used for the feature extraction step, since only extracted features are compared instead of whole images. In our work, images are normalized using z-score normalization and encoded using the Resnet-50 pretrained MedicalNet model (He et al., 2015). The dimensions of the resulting feature map is further reduced using an adaptive average pooling layer to a final size of (2048x4x4x4). This embedding is flattened to a feature vector and compared using the RMSE and the cosine similarity.

**Cosine similarity**

The cosine similarity was calculated after flattening the 3D images to 1-dimensional vectors. The numpy library was used for calculation with the following formula.

[**
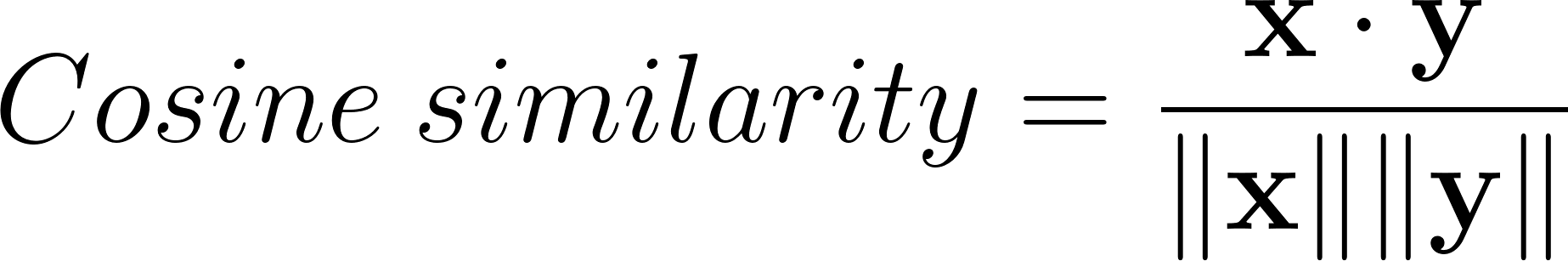
**](https://www.codecogs.com/eqnedit.php?latex=%7BCosine%5C%3Asimilarity%7D%20%3D%20%5Cfrac%7B%5Cmathbf%7Bx%7D%20%5Ccdot%20%5Cmathbf%7By%7D%7D%7B%5C%7C%20%5Cmathbf%7Bx%7D%20%5C%7C%20%5C%7C%20%5Cmathbf%7By%7D%20%5C%7C%7D#0)

where x . y is the dot product and ||x|| denotes the euclidean norm.

**Segmentation-level Analysis**

Different medical imaging modalities contain target structures relevant for diagnosis and treatment decisions. For instance, the first use case of this study includes NCCT images of patients with intracerebral hemorrhage. The hemorrhage lesions constitute the region of interest and are more salient for the diagnosis and treatment decisions compared to other parts of the image. Generative models trained on data containing a significant region of interest (ROI) such as hemorrhage lesions should preserve and capture the predictive properties of the real images with respect to the pathology. Therefore, segmentations of ROIs should play a role in replica detection frameworks. Similar ROIs can argue in favour of a generated image being a replica, although there might be considerable differences in the background. In our work, we use the Dice coefficient (Zou et al., 2004) and the average surface distance (ASD) from an open-source implementation to compare segmentations (https://github.com/google-deepmind/surface-distance).

**Dice coefficient**

The Dice coefficient is arguably the most popular measure for segmentation performance assessment. We use the Dice coefficient to perform a region of interest level analysis by comparing segmentations of the leading structures in generated and real images. The Dice coefficient can be calculated using the confusion matrix values of true positives (TP), false positives (FP) and false negatives (FN).

[
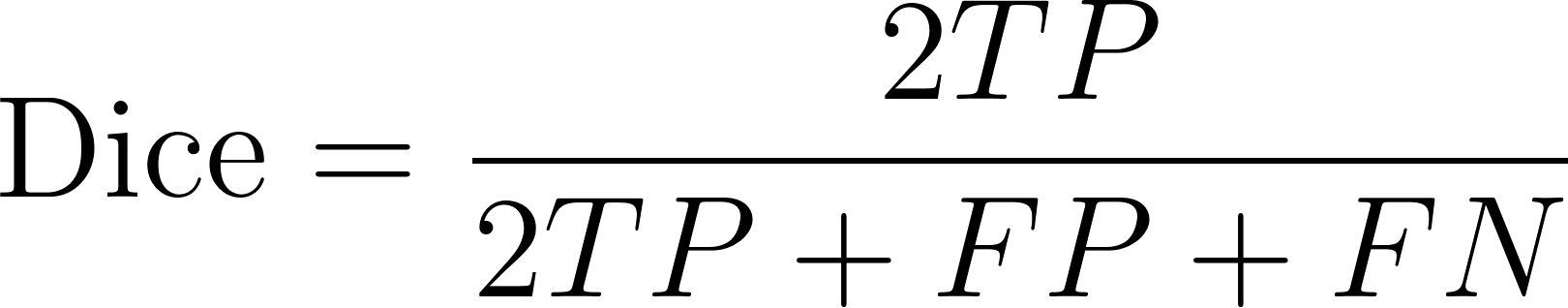
](https://www.codecogs.com/eqnedit.php?latex=%5Ctext%7BDice%7D%20%3D%20%5Cfrac%7B2TP%7D%7B2TP%20%2B%20FP%20%2B%20FN%7D#0)

**Average Surface Distance**

The average surface distance is the average distance of outline of the predicted surface to the outline of the ground truth surface and vice versa.

Let S1, S2 be two surfaces and let d(s, S) be the distance from voxel s to surface the S. The distance is defined as


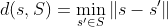


then the average surface distance ASD can be defined as:


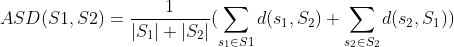


In the exception case for the multiclass evaluation, where one class has an empty segmentation, the 95 th percentile Hausdorff distance of the whole image was used as the ASD value for that class.

For our replica detection framework, we use an open source implementation of ASD from (<https://github.com/google-deepmind/surface-distance>) due to its popularity and ease of integration. Other distance based measures might be used interchangeably to assess similarity between segmentations in the scope of replica detection.

**Table 2.** Replica detection methods overview and runtime analysis.

| **Measure** | **Analysis Level** | **Runtime** | |
| --- | --- | --- | --- |
|  |  | **NCCT** | **TOF-MRA** |
| **Mean Absolute Error (MAE)** | **Image-level** | 16 mins | 2 mins |
| **Root Mean Square Error (RMSE)** | **Image-level** | 16 mins | 2 mins |
| **SSIM** | **Image-level** | 76 mins | 11 mins |
| **Embeddings**  **RMSE** | **Feature-level** | 4 mins | 2 mins |
| **Embeddings**  **Cosine similarity** | **Feature-level** | 4 mins | 2 mins |
| **Dice (binary)** | **Segmentation-level** | 23 mins | 3 mins |
| **Dice (multiclass)** | **Segmentation-level** | - | 4 mins |
| **Average surface distance (binary)** | **Segmentation-level** | 31 mins | 16 mins |
| **Average surface distance (multiclass)** | **Segmentation-level** | **-** | 16 mins |

**B. Full replica detection plots**


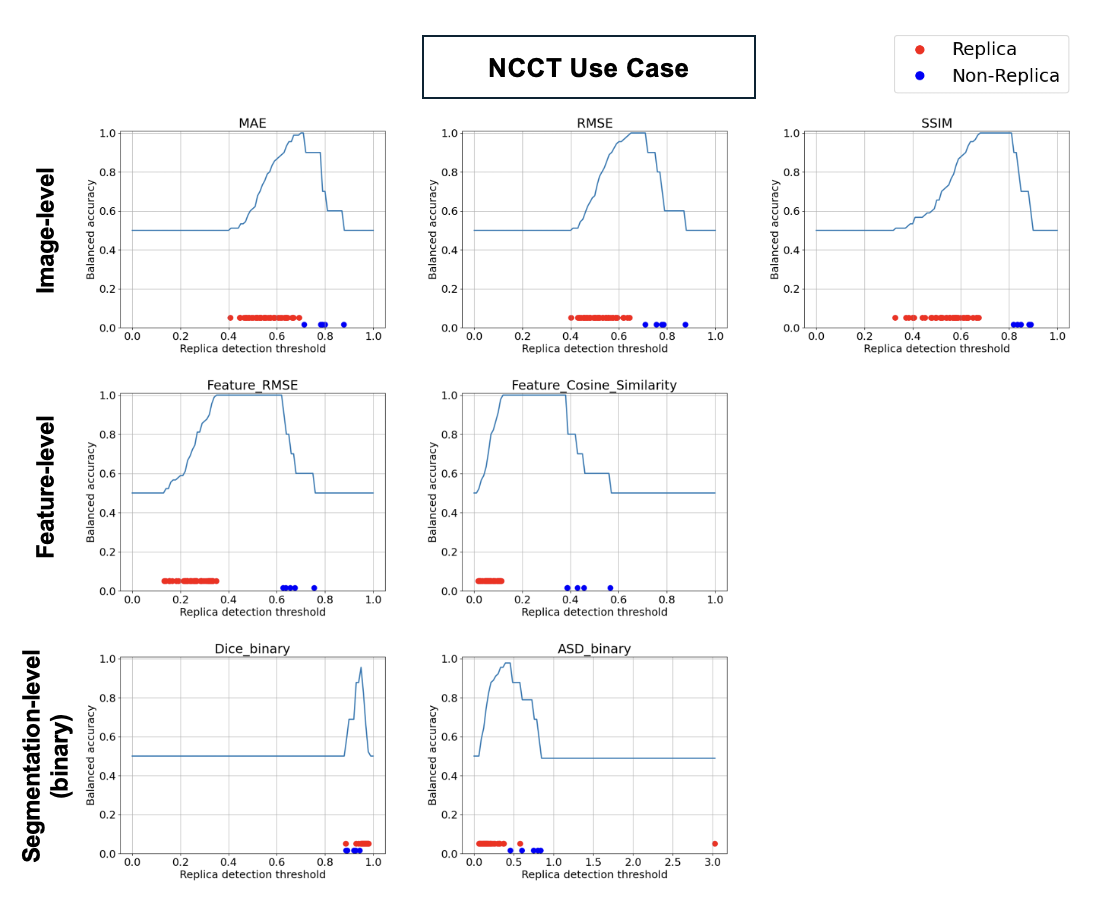


**
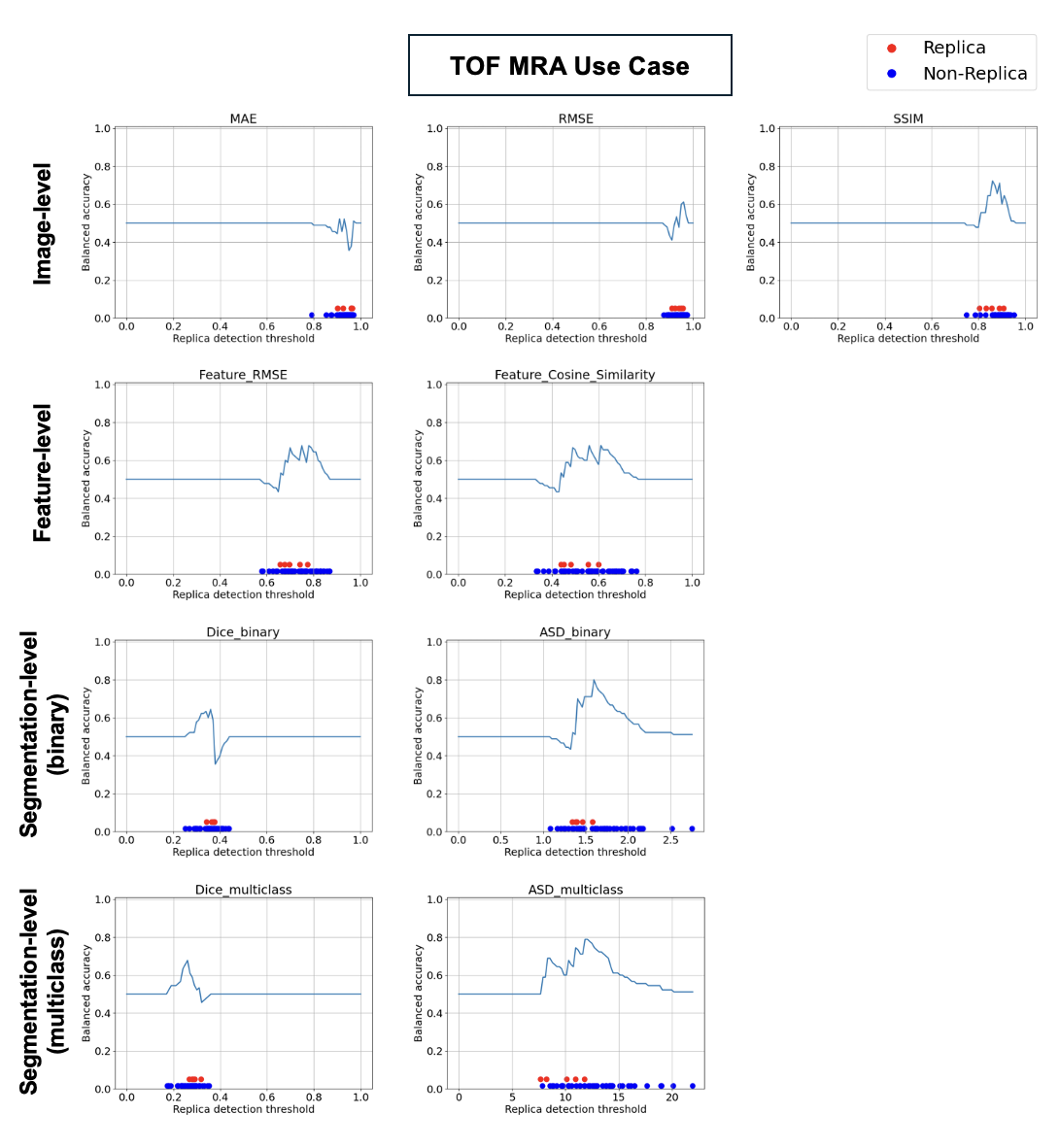
**

**References**

He, K., Zhang, X., Ren, S., & Sun, J. (2015). Deep residual learning for image recognition

(arXiv:1512.03385). arXiv. https://doi.org/10.48550/arXiv.1512.03385

Jush, F. K., Vogler, S., Truong, T., & Lenga, M. (2024). Content-based image retrieval for

multi-class volumetric radiology images: A benchmark study (arXiv:2405.09334). arXiv.

https://doi.org/10.48550/arXiv.2405.09334

Tak, D., Garomsa, B. A., Chaunzwa, T. L., Zapaishchykova, A., Climent Pardo, J. C., Ye,

Z., Zielke, J., Ravipati, Y., Vajapeyam, S., Mahootiha, M., Smith, C., Familiar, A. M., Liu,

K. X., Prabhu, S., Bandopadhayay, P., Nabavizadeh, A., Mueller, S., Aerts, H. J., Huang, R.

Y., … Kann, B. H. (2024). A foundation model for generalized brain MRI analysis.

https://doi.org/10.1101/2024.12.02.24317992

Zhou, W., & Bovik, A. C. (2009). Mean squared error: Love it or leave it? A new look at

signal fidelity measures. IEEE Signal Processing Magazine, 26(1), 98–117.

https://doi.org/10.1109/MSP.2008.930649

Chen, S., Ma, K., & Zheng, Y. (2019). Med3D: Transfer learning for 3D medical image

analysis. ArXiv. ArXiv:1904.00625. http://arxiv.org/abs/1904.00625

#

#

# 
